# Penny-A-Week Charity

**Published:** 1903-07-25

**Authors:** 


### PENNY-A-WEEK CHARITY.

The Bradwell and Wolverton "Good Samaritan Society" does a work which might with advantage be copied by other rural districts. By far the larger part of its funds are collected in penny-a-week subscriptions from a body of 1,900 members. The contributions thus gathered amount to £407 out of a total income of £425, and represent the collection of nearly 99,000 pennies. On this sum the collectors receive 10 per cent. commission, but when one considers the labour involved in the ingathering of weekly contributions, this honorarium cannot be considered excessive payment. The money thus collected is spent in various ways. In the first place money is laid out in the purchase, as needed, of letters of admission to several hospitals and convalescent homes. The Northampton General Hospital is the one most used, but in the interests of subscribers the society also subscribes for letters to the University College Hospital, the Brompton Hospital for Consumption, the National Hospital for the Paralysed and Epileptic, Queen Square, the Birmingham Eye Hospital, the Bedford County Hospital, and several others. To many of these it gives, besides the subscriptions necessary for the obtaining of such letters as are required, donations of varying amounts, thus recognising the principle that in hospital matters the sums necessary to obtain letters of admission does not represent the whole of the beneficiaries' indebtedness in the matter. Besides this, the committee lend out to sick people who are being nursed at their own homes medical appliances of various kinds. Among these are air cushions, air pillows, bed rests, bronchitis kettles, bed cradles, hot-water bottles, inhalers, ice bags, ice caps, baths, crutches, feeding cups, enemas, and thermometers. Bath chairs also are available for the use of invalids, besides a spinal chair and a water bed. The committee has made arrangements for supplying ice to sick persons at short notice, and in necessitous cases it gives, on presentation of a proper medical certificate, trusses, belts, and elastic stockings. District nurses know how often such things are needed in their work, and frequently how difficult it is to obtain them. Private charity may occasionally supply some of them, but in many cases makeshifts have to be contrived. The benefits of the society are not confined to members, though, as these are requested to mention the fact, and the name of their collector when asking assistance of any kind, we may infer that they have—very reasonably—a preferential claim on it. A medical certificate may be demanded of applicants for help, and they are warned in the society's report that "it must be distinctly understood that the society's object is to supplement the treatment of the local medical men rather than to forestall it." In short, this society seems to be one which performs a most useful mission, and which could, if the officials and collectors were earnest and energetic, be carried on in any neighbourhood in town and country. It is quite worth the consideration of working men, who, if they undertook the work, would be able to carry it out better than outsiders. In very few cases would they find the penny-a-week subscription grudged, if it were collected weekly. But the regular weekly visit of the collector is an essential feature of the scheme.

